# Inner Engineering Practices and Advanced 4-day Isha Yoga Retreat Are Associated with Cannabimimetic Effects with Increased Endocannabinoids and Short-Term and Sustained Improvement in Mental Health: A Prospective Observational Study of Meditators

**DOI:** 10.1155/2020/8438272

**Published:** 2020-06-05

**Authors:** Senthilkumar Sadhasivam, Suresh Alankar, Raj Maturi, Ramana V. Vishnubhotla, Mayur Mudigonda, Dhanashri Pawale, Santhosshi Narayanan, Sepideh Hariri, Chithra Ram, Tracy Chang, Janelle Renschler, George Eckert, Balachundhar Subramaniam

**Affiliations:** ^1^Department of Anesthesia, Indiana University School of Medicine, 1130 West Michigan St., Fesler Hall 204, Indianapolis, IN 46202, USA; ^2^University of Louisville, 201 Abraham Flexner Way, Louisville, KY 40202, USA; ^3^Department of Ophthalmology, Indiana University School of Medicine, 1160 W. Michigan St., Eugene and Marilyn Glick Eye Institute, Indianapolis, IN 46202, USA; ^4^Independent Researcher, Philadelphia, PA 19103, USA; ^5^Lawrence Berkeley National Laboratory, 1 Cyclotron Road, Berkeley, CA 94720, USA; ^6^Department of Palliative, Rehabilitative and Integrative Medicine, The University of Texas, MD Anderson Cancer Center, Unit 1465, 1400 Pressler St., Houston, TX 77030, USA; ^7^Department of Physics, Seattle University, 901 12^th^ Ave., Seattle, WA 98122, USA; ^8^Department of Radiology, University of Louisville Hospital, 530 S. Jackson St., CCB-C07, Louisville, KY 40202, USA; ^9^Department of Labor Studies and Employment Relations, School of Management and Labor Relations, Rutgers University, 50 Labor Center Way, New Brunswick, NJ 08901, USA; ^10^Department of Biostatistics, Indiana University School of Medicine, 410 W. 10th St., HITS 3000, Indianapolis, IN 46202, USA; ^11^Center for Anesthesia Research Excellence, Department of Anesthesia, Critical Care and Pain Medicine, Beth Israel Deaconess Medical Center, 375 Longwood Ave., Boston, MA 02215, USA

## Abstract

**Background:**

Anxiety and depression are common in the modern world, and there is growing demand for alternative therapies such as meditation. Meditation can decrease perceived stress and increase general well-being, although the physiological mechanism is not well-characterized. Endocannabinoids (eCBs), lipid mediators associated with enhanced mood and reduced anxiety/depression, have not been previously studied as biomarkers of meditation effects. Our aim was to assess biomarkers (eCBs and brain-derived neurotrophic factor [BDNF]) and psychological parameters after a meditation retreat.

**Methods:**

This was an observational pilot study of adults before and after the 4-day Isha Yoga Bhava Spandana Program retreat. Participants completed online surveys (before and after retreat, and 1 month later) to assess anxiety, depression, focus, well-being, and happiness through validated psychological scales. Voluntary blood sampling for biomarker studies was done before and within a day after the retreat. The biomarkers anandamide, 2-arachidonoylglycerol (2-AG), 1-arachidonoylglycerol (1-AG), docosatetraenoylethanolamide (DEA), oleoylethanolamide (OLA), and BDNF were evaluated. Primary outcomes were changes in psychological scales, as well as changes in eCBs and BDNF.

**Results:**

Depression and anxiety scores decreased while focus, happiness, and positive well-being scores increased immediately after retreat from their baseline values (*P* < 0.001). All improvements were sustained 1 month after BSP. All major eCBs including anandamide, 2-AG, 1-AG, DEA, and BDNF increased after meditation by > 70% (*P* < 0.001). Increases of ≥20% in anandamide, 2-AG, 1-AG, and total AG levels after meditation from the baseline had weak correlations with changes in happiness and well-being.

**Conclusions:**

A short meditation experience improved focus, happiness, and positive well-being and reduced depression and anxiety in participants for at least 1 month. Participants had increased blood eCBs and BDNF, suggesting a role for these biomarkers in the underlying mechanism of meditation. Meditation is a simple, organic, and effective way to improve well-being and reduce depression and anxiety.

## 1. Introduction

In the modern world, stress and anxiety have become synonymous with success and productivity. Over the course of a lifetime, almost half of Americans are estimated to have a mental disorder such as anxiety (>25% incidence) or mood disorder (>20% incidence) [1, 2]. There is increasing interest and accessibility of nonpharmaceutical treatment options for these disorders, such as meditation, counseling, and lifestyle changes (e.g., diet, regular exercise). Meditation is increasingly being recognized as a simple and effective tool to decrease perceived stress and increase general well-being [3].

Although certain benefits of meditation have been widely acknowledged, the physiological basis and underlying mechanisms have not been well-characterized. Cortisol levels may be reduced [4–7], and the neuroregulator brain-derived neurotrophic factor (BDNF) has been shown to increase with meditative practices [6, 8–10]. Following a 3-month Isha yoga and meditation retreat, participants had nearly a 3-fold increase in mean BDNF. Increased BDNF signaling, and thus enhanced neurogenesis and/or neuroplasticity, was associated with improved resilience and well-being. Decreases in inflammatory biomarkers, including interleukin- (IL-) 6, C-reactive protein and tumor necrosis factor- (TNF-) *α*, and increases in *β*-endorphins have also been shown with various meditation/yoga practices [11].

Endocannabinoids (eCBs) are lipid mediators found in the brain and peripheral tissues that mimic the action of Δ^9^ -tetrahydrocannabinol (THC) [12]. The eCBs N-arachidonoylethanolamine (AEA or anandamide) and 2-arachidonoylglycerol (2-AG) have been associated with enhanced mood and feelings of blissfulness. The term “anandamide” was even derived from Sanskrit (“ananda” meaning bliss) for its cannabimimetic effects. Other eCBs include 1-arachidonoylglycerol (1-AG), docosatetraenoylethanolamide (DEA), and oleoylethanolamide (OLA) [12]. Serum levels of both anandamide and 2-AG may be reduced in patients with depression and anxiety, and the eCB system plays a fundamental role in emotional homeostasis [13]. We hypothesized that eCBs may be involved in the underlying mechanisms leading to the beneficial effects of meditation.

To gain greater insights into the psychological and physiological effects of meditation, we evaluated participants at Bhava Spandana Program (BSP), an experiential program leading to deep states of meditativeness in just a short period of time. In Sanskrit, “Bhava” means “sensation” and “Spandana” can be loosely translated as “resonance.” Essentially, it means “resonance with life.” The program [14] is a 4-day advanced yoga and meditation retreat held at the Isha Institute of Inner Sciences, located in Tennessee in the USA. The prerequisite for participating in the BSP is completion of the Inner Engineering program offered worldwide. The Inner Engineering program teaches participants a 21-minute yoga and meditation practice called Shambhavi Mahamudra [15], an Inner Engineering Level 1 program, which reduces stress and improves well-being [3]. Other Isha Yoga meditation programs have shown benefits including increased gamma brainwave amplitude, heart rate variability, sympathovagal balance, BDNF, and cortisol awakening response [16–18].

The BSP is a 4-day, 3-night, residential program offered to those who have been initiated into Shambhavi Mahamudra. This advanced meditation program is designed to provide the opportunity to go beyond the limitations of body and mind and experience higher levels of consciousness. Bhava Spandana offers the experience of a world of unbounded love and joy.

Through powerful processes and meditations, BSP creates an intensely energetic situation, where individuality and the limitations of the5 sense organs can be transcended, creating an experience of oneness and resonance with the rest of existence. Humans have lived within the limitations of human senses. Bhava Spandana is like giving one a lift or jump over the wall to have a peep at life beyond the limitations of the five sense perceptions. Past BSP participants have described feelings of blissfulness, ecstasy, intense happiness, inclusive perception, and higher states of consciousness during and after the program.

Our hypothesis was that BSP meditation would significantly reduce depression and anxiety and improve happiness and positive well-being in the short-term as well as in the long-term. Furthermore, these changes in psychological well-being are associated with objective changes in blood eCB and BDNF levels. This study specifically aimed (a) to assess the impact of this 4-day guided, experiential meditation retreat on mental health and well-being (happiness, awareness, well-being, anxiety, and depression), (b) to correlate reported psychological changes with objective blood biomarkers (eCBs and BDNF) immediately after the retreat, and (c) to assess any persistent impact on participants' psychological well-being one month after the retreat.

## 2. Materials and Methods

### 2.1. Study Population

In October 2017, adult participants (≥18 years) were registered to participate in the 4-day BSP meditation program at Isha Institute of Inner-Sciences, McMinnville, Tennessee, USA. All registrants received an e-mail which invited them to participate in an online survey 2 weeks before the meditation program. Participation in the study was voluntary. Individuals who were unable to read and/or comprehend the consent forms were excluded. For the blood biomarker subset of the study, individuals were excluded if they reported use of active marijuana, opioid, or other illegal drugs, or if they were taking antidepressant medication. The protocol was reviewed and approved by Institutional Review Board of Indiana University School of Medicine. All participants gave electronic informed consent.

### 2.2. Meditation Program

The BSP is a 4-day advanced yoga and meditation program designed to enhance participants' perception and sensitivity to life by going beyond the limitations of body and mind and experiencing higher levels of consciousness [14]. Participants are required to complete an online or in-person prerequisite program (Inner Engineering) that includes Shambhavi Mahadmudra kriya yoga practice [15].

### 2.3. Surveys

Participants completed the preprogram (“baseline”) survey within 2 weeks before the retreat and postprogram (“immediately after retreat”) survey within 2 weeks after the retreat, and a 1-month follow-up survey. These surveys included scales that have well-established reliability and validity in the literature. Depression is measured by the 20-item Center for Epidemiologic Studies Depression Scale [19] (CES-D). A sample item is, “During the past week, I was bothered by things that usually do not bother me.” The response is coded from 0 (rarely) to 3 (most of the time) [20]. The CES-D composite score is the sum of 20 scores. The possible range is 0 to 60. If more than 4 questions are unanswered, no score is assigned. A score of 16 points or higher is considered depression.

Anxiety is measured by the 8-item Patient-Reported Outcomes Measurement Information System (PROMIS) Emotional Distress-Anxiety (short form) [21]. The scale uses a 7-day time frame and a sample item is, “I felt fearful.” The response is coded on a 5-point scale from 1 (never) to 5 (always) [21].

Mindfulness is measured by the 15-item Mindful Attention Awareness-Trait Scale (MAAS) [22]. The MAAS is designed to assess awareness and observation of what is occurring in the present moment in participants' everyday experience. A sample statement is, “I could be experiencing some emotion and not be conscious of it until sometime later.” The response is coded on a 6-point scale from 1 (almost always) to 6 (almost never). The MAAS score is computed as the average of 15 items.

Well-being is measured by Ryff's 42-item Psychological Well-Being (PWB) Scale [23]. Ryff's PWB is a theoretically grounded instrument that encompasses multiple facets of psychological well-being, autonomy, environmental mastery, personal growth, positive relations, purpose of life, and self-acceptance. Since the inception of the scale thirty years ago, the scale has been translated into 30 languages. A sample item for the autonomy is, “I am not afraid to voice my opinions, even when they are in opposition to the opinions of most people.” A sample item for environmental mastery is, “In general, I feel I am in charge of the situation in which I live.” A sample item of personal growth is, “I am not interested in activities that will expand my horizons.” A sample item of positive relations is, “Most people see me as loving and affectionate.” A sample item of purpose of life is, “I live life one day at a time and do not really think about the future.” A sample item of self-acceptance is, “When I look at the story of my life, I am pleased with how things have turned out.” For each item, the response is coded on a 6-point Likert scale from 1 (strongly disagree) to 6 (strongly agree). The composite score of each subscale is the sum of its own 7 items and the composite score of the global scale is the sum of 42 items. The scale has been used in a longitudinal follow-up of the US national sample [23]. Happiness is a global 0–10 happiness scale.

Only those who completed the first survey were asked to complete the postintervention surveys. Those who did not complete the survey within a week of the first invitation were sent e-mail reminders.

### 2.4. Blood Sampling

Survey participants were given the option for blood biomarker evaluation. Informed consent for blood draws was obtained, and the participant's samples were collected at the following time points: (1) preprogram (up to 2 days before program began) and (2) postprogram (within 2 days after program ended). Standard sterile venipuncture technique was used to collect 10 mL of blood divided into different sampling tubes for measuring anandamide, 1-AG, 2-AG, DEA, OLA, and BDNF.

For participant confidentiality, samples were deidentified with a code for sharing with individuals outside of the study team. The biomarker samples were sent immediately to the analysis lab in Denver, Colorado. As anandamide is quickly catabolized by Fatty Acid Amide Hydrolase (FAAH), the fresh blood samples were centrifuged and extracted plasma was frozen immediately to preserve and accurately measure this biomarker.

### 2.5. Data Analysis

Repeated measures analysis of variance (ANOVA) was used to compare preprogram (baseline), immediately after program, and 1-month follow-up survey responses; an unstructured variance-covariance matrix was used. In the subset of subjects with biomarkers assessed, paired *t*-tests were used to compare biomarkers for significant change from before meditation to after meditation.

Pearson correlation coefficients were used to evaluate the associations between the changes in biomarkers and changes in psychological states. In addition, the change in biomarkers was categorized into two groups, including one group where there was at least a 20% increase and another where there was less than 20% increase. The responses to the psychological scale were separately analyzed for both of these groups. A 5% significance level was used for all tests.

## 3. Results

### 3.1. Study Participation

Three hundred forty-eight participants completed the preprogram survey and 323 participants completed the postprogram survey, resulting in a 93% response rate. The 1-month follow-up survey was completed by 308 participants. One hundred and forty-two participants volunteered for blood sampling for biomarker assays before and after meditation retreat. Subject characteristics are shown in Table 1.

### 3.2. Markers of Well-Being

Scores for depression (CES-D) and anxiety significantly decreased from baseline to immediately after retreat (*P* < 0.001; Table 2). The mean CES-D score was relatively low at baseline (10.3) and declined by 26%, mean decrease of 2.7 (SD = 8.6), and effect size of 0.31. The mean standardized anxiety score was also low at baseline and declined by 23% at the immediate postretreat survey, mean decrease of 0.46 (SD = 0.76), and effect size of 0.60.

**Figure 1:**
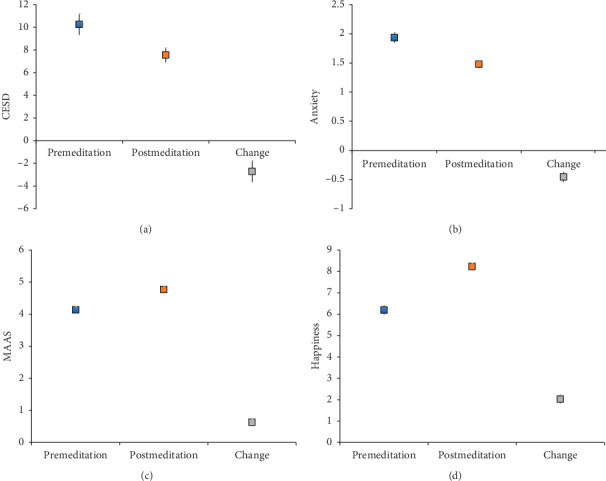
Mean with 95% confidence interval for depression (CESD), anxiety, focus (MAAS), and happiness at premeditation, postmeditation, and change (post-pre). Focus and happiness increased significantly while depression and anxiety decreased significantly (all *P* < 0.001). CESD = Center for Epidemiological Studies Depression Scale; MAAS = mindful attention and awareness scale.

Scores for mindfulness (MAAS), happiness, and psychological well-being (PWB) increased significantly from baseline to immediately after retreat (*P* < 0.001; Table 2, Figure 1). Mean (SE) increases and effect sizes were 0.63 (0.85) and 0.75 for mindfulness, 2.0 (1.8) and 1.1 for happiness, and 15.7 (20.8) and 0.75 for well-being. These improvements were maintained at the 1-month follow-up. The autonomy measure of PWB showed a continuous improvement, as PWB scores increased even more on the 1-month survey compared with immediately after retreat (*P* = 0.003; Table 2). All of these primary psychological changes were statistically significant even after adjusting for multiple testing.

Cronbach's alpha values for the internal consistency reliability of the psychological scales were all acceptable: CESD = 0.90; anxiety = 0.94; mindfulness = 0.93; PWB = 0.92.

### 3.3. Biomarkers

**Figure 2:**
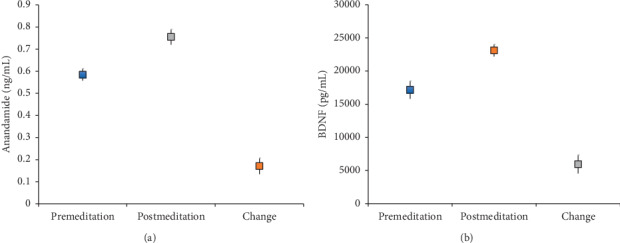
Mean with 95% confidence interval for anandamide (ng/mL) and BDNF (pg/mL) at premeditation, postmeditation, and change (post-pre). Both biomarkers increased significantly (*P* < 0.001). BDNF = brain-derived neurotrophic factor.

Endocannabinoids (anandamide, 2-AG, 1-AG, DEA, and OLA) and BDNF were analyzed in 142 participants. Compared with baseline levels, all immediately after retreat biomarker levels were higher (Table 3, Figure 2). Some individuals had multiple-fold increases in anandamide, and the effect size for biomarker changes ranged from 0.17 to 0.92. High interindividual variability was observed among participants. Specifically, 2-AG increased significantly (*P* < 0.001) by 2.0 ng/mL (SD = 2.8) with an effect size of 0.71, and 71% of participants had an increase of at least 20%. Similarly, BDNF increased (*P* < 0.001) by 5945 pg/mL (SD = 8414) with an effect size of 0.71, and 53% of participants had an increase of at least 20%.

Participants with increases in 2-AG levels larger than 20% after meditation from the baseline had larger increases in mindfulness, happiness, and positive well-being (total, autonomy, environmental mastery, positive relations, and self-acceptance) scores (Supplementary Table (available here)). Similarly, participants with increases in 1-AG or total AG levels larger than 20% had larger increases in happiness and positive well-being (total and self-acceptance for 1-AG, positive relations for total Ag; Supplementary Table).

## 4. Discussion

In this large, prospective study, we showed that a short, intense guided experiential meditation (BSP) significantly decreases anxiety and depression and improves psychological well-being, happiness, and mindfulness and that these improvements sustain for at least a month. For the first time, we showed that objective blood biomarkers, eCBs (anandamide, 1-AG, 2-AG, DEA, and OLA) and BDNF, all increased following an advanced meditation, BSP, suggesting a role for these mediators in the underlying mechanisms of meditation. The specific mechanisms linking meditation to positive psychological effects have not been well-characterized in the literature, and this research provides early evidence that the eCB system and BDNF are significantly increased after advanced meditation.

Low blood levels of anandamide and 2-AG have been reported in patients with depression [24]. Deficiencies in anandamide can be associated with acute stress [25] and increased pain [26]. Studies have related depression and anxiety to the expression and/or functionality of cannabinoid type 1 (CB1) receptors and FAAH in brain areas belonging to the amygdala-hippocampal-corticostriatal neural circuit, especially the frontal cortex in depression and the amygdala in anxiety disorders [13]. Increasing patients' anandamide levels is a potential solution for many ailments including depression [27, 28], fibromyalgia [29, 30], inflammatory bowel disease [31–33], and cancer [34, 35]. Our findings that meditation can increase blood eCB levels and improve well-being suggest that meditation might be further explored as a treatment modality for anxiety and depression and possibly even other diseases.

The level of increase in eCBs following meditation was large enough to be deemed clinically significant, as effect sizes ranged from 0.77 to 0.90, and some participants had 2-3-fold increases. This is more than reported eCB increases associated with moderate intensity aerobic physical exercise [36] and sexual orgasm [37]. Modest increases (<40%) in eCBs explain pleasurable effects of singing and exercise and ultimately some of the long-term beneficial effects on mental health, cognition, and memory [38]. Moreover, increases in eCB levels with moderate exercise have been associated with mood improvements in patients with major depressive disorders, explaining the biological mechanism behind mood enhancement with exercise in major depressive disorders [39] and posttraumatic stress disorder [40].

Activation of CB1 receptors has been shown to increase BDNF expression [41]. We demonstrated increased BDNF levels following meditation, and our findings validate similar observations from a previous Isha mediation program [18]. This is notable as BDNF is associated with neuronal regeneration [42–45]. BDNF deficiencies have been linked to mental disorders such as depression [46, 47], bipolar disorder [46], and schizophrenia [48, 49]. While THC has been shown to increase BDNF levels, this effect is mitigated by even light chronic cannabis use [50]. Therefore, endogenous activation of CB1 receptors through other ways, such as meditation, would be necessary to have a long-lasting impact on BDNF expression. Future studies might examine the efficacy of meditation as a therapy for bipolar disorder, schizophrenia, and other mental health conditions.

The sustained positive impacts from meditation, even one month after BSP, are encouraging. The BSP participants experienced persistent positive benefits from the short and intensive BSP meditation, extending beyond an immediate “high” or bliss feeling from increased eCBs. An intense short-term negative experience can lead to diseases like posttraumatic stress disorder; thus an intense short-term positive experience might similarly lead to lasting positive psychological consequences. Completion of Inner Engineering program (level 1) and daily practice of Shambhavi kriya is a requirement for BSP, and participants were encouraged to continue this daily practice after the BSP to maximize potential benefits. Continued daily meditation may have contributed to some of the sustained positive results at 1 month after BSP in our study. Given certain logistical constraints (some BSP participants were from different states or other countries), we did not collect blood samples to reassess biomarkers at 1 month. This could be evaluated in future studies.

Weak but positive correlations between biomarker changes and changes in psychological measures, including some interindividual variations, were observed in this study. The findings suggest that there are interindividual variations in objective biomarkers, similar to variation in experiences and happiness levels. Because of the disparity in subjective experience and physiological responses, the benefits and expression of biomarkers from meditation may vary between individuals. This needs further study to explore the interindividual variations in subjective and biomarker responses in people simultaneously participating in a meditation program. Factors such as genetics/epigenetics, gene expression, and corresponding psychological health may explain these interindividual variations.

Strengths of this study are prospective design, relatively large number of meditators participating simultaneously in validated psychological surveys and blood biomarkers before and after meditation, hypothesis-driven approach to mechanistically associate the beneficial effects of meditation with simultaneous objective biomarker assay associations, use of validated scales for sustained psychological benefits of meditation for up to 1 month, and generalizability of the findings as the study meditators had diversity in terms of age, sex, race, and ethnicity. Limitations included lack of 1-month postretreat biomarker assessment, as discussed above. Additionally, the 4-day BSP retreat had multiple daily meditation sessions, and the “immediately-after-meditation” blood samples were actually collected after the entire program. Because eCBs (particularly anandamide) are highly labile, biomarker expression would likely have been even higher if samples had been collected during the retreat. The study population had low baseline levels of anxiety and depression; therefore, differing results might be obtained by analyzing the effects of meditation on patients with psychological disorders. Finally, participants were not surveyed on postretreat meditation or other activities that may have contributed to the sustained long-term benefits.

Since the meditators in this study were prospectively and objectively followed over 3 time points, each individual essentially served as his/her own control. To rule out a placebo effect, we analyzed objective biomarkers in addition to self-reported psychological changes. The lack of a separate, matched control group may be viewed as a study limitation. However, the significant changes in objective biomarkers and the sustained psychological benefits (despite only a brief meditation retreat) strongly support that this is a true effect rather than a placebo. This pilot study provides evidence to support future larger studies (including a control group) with longer-term follow-up to assess the persistent benefits of meditation.

More research is needed to understand the role of the eCB system in the mechanisms of meditation. In future, the effects of meditation may be replicated through pharmacologic intervention, as FAAH inhibitors can reduce anandamide degradation. Inhibition of FAAH has been considered as a treatment option for anxiety and other psychological disorders [25, 51–54]; however, a clinical trial of one compound in 2016 caused severe adverse effects including one death [55]. Meditation remains a simple and safe option to improve well-being and benefit individuals with depression, anxiety, and other disorders.

## 5. Conclusion

An intense 4-day guided Isha meditation retreat significantly decreased depression and anxiety while improving happiness, mindfulness, and psychological well-being. Increased blood endocannabinoids and BDNF were also observed immediately after the intense meditation, potentially explaining the underlying mechanism behind the improvements in happiness and other psychological benefits. The psychological effects of the meditation retreat were sustained for at least 1 month. Meditation might serve as a simple, organic, nonpharmacological, and effective low-risk therapy or a prevention strategy for depression and anxiety, while improving happiness and well-being. Future studies are needed to investigate the role of the eCB system as mediators of the positive effects of meditation, the sustained benefits of meditation over longer-term, and reasons for interindividual variability in meditation response.

## Figures and Tables

**Table 1:** Subject characteristics.

Race/ethnicity	*n* (%)
Asian: Far Eastern	7 (2%)
Asian: Indian	243 (70%)
Asian: Middle Eastern	6 (2%)
Black (African American)	6 (2%)
Hispanic	14 (4%)
White Nonhispanic (Caucasian)	57 (16%)
Mixed race: Asian-White	1 (<1%)
Mixed race: others	4 (1%)
Other	5 (1%)
I prefer not to answer	5 (1%)
Gender	
Female	172 (49%)
Male	176 (51%)
Chronic medical or psychological disease	
No	260 (75%)
Yes	85 (25%)
Medications	
None	308 (89%)
Yes	40 (11%)
Marijuana use in the last 6 months	
No	319 (92%)
Yes	29 (8%)
Alcohol use	
No	210 (60%)
Yes	138 (40%)
Regular exercise	
No	122 (35%)
Yes	226 (65%)

**Table 2:** Results of psychological surveys before and after a 4-day Bhava Spandana Program meditation retreat.

Psychological parameter	Baseline before retreat		Immediately after retreat		1-month after retreat	
	*N*	Mean (SE)	*N*	Mean (SE)	*N*	Mean (SE)
CES-D (depression)	348	10.26 (0.47)	323	7.55 (0.32)^a^	308	7.67 (0.37)^a^
Anxiety scale	342	1.93 (0.04)	312	1.48 (0.03)^a^	300	1.54 (0.04)^a^
MAAS (mindfulness)	346	4.14 (0.05)	320	4.77 (0.05)^a^	301	4.80 (0.05)^a^
Happiness score	342	6.20 (0.10)	312	8.23 (0.08)^a^	291	7.62 (0.09)^a^
PWB (well-being) total	342	187.3 (1.5)	312	203.0 (1.3)^a^	293	203.4 (1.4)^a^
PWB autonomy		30.08 (0.34)		33.14 (0.32)^a^		33.93 (0.31)^a,b^
PWB environmental mastery		28.49 (0.28)		30.88 (0.25)^a^		30.99 (0.26)^a^
PWB personal growth		35.23 (0.28)		37.18 (0.23)^a^		37.27 (0.25)^a^
PWB positive relations		31.99 (0.34)		34.91 (0.30)^a^		34.73 (0.32)^a^
PWB purpose in life		30.30 (0.30)		32.12 (0.27)^a^		31.85 (0.28)^a^
PWB self-acceptance		31.20 (0.38)		34.79 (0.31)^a^		34.77 (0.33)^a^

^a^Significantly different from baseline value (*P* < 0.001), ^b^Significantly different from immediately after-retreat value (*P*=0.003). All changes were statistically significant even after adjusting for multiple testing. CES-D = Center for Epidemiologic Studies Depression; MAAS = Mindful Attention and Awareness Scale; *N* = number of participants; PWB = psychological well-being; SE = standard error.

**Table 3:** Changes in blood biomarkers (endocannabinoids and BDNF) following a 4-day Bhava Spandana Program meditation retreat.

Biomarker	*N*	Baseline before retreat	Immediately after retreat	Change	*P* value	Effect size	Participants (%) with >20 (%) increase
		Mean (SD)	Mean (SD)	Mean (SD)			
Anandamide (ng/mL)	142	0.58 (0.16)	0.75 (0.21)	0.17 (0.22)	<0.001	0.77	59
2-AG (ng/mL)	142	2.2 (1.6)	4.2 (3.1)	2.0 (2.8)		0.71	71
1-AG (ng/mL)	142	4.4 (3.3)	14.3 (11.3)	9.8 (10.6)		0.92	92
Total AG (ng/mL)	142	6.6 (4.8)	18.5 (14.2)	11.9 (13.2)		0.90	89
DEA (ng/mL)	142	0.21 (0.07)	0.27 (0.09)	0.06 (0.07)		0.86	60
OLA (ng/mL)	142	22.4 (36.5)	30.9 (45.4)	8.5 (50.7)	0.048	0.17	52
BDNF (pg/mL)	142	17152 (8048)	23097 (5526)	5945 (8414)	<0.001	0.71	53

1-AG = 1-arachidonoylglycerol (degradation product of 2-arachidonoylglycerol); 2-AG = arachidonoylglycerol (unstable in plasma without pH adjustment); total AG = sum of 1-arachidonoylglycerol and 2-arachidonoylglycerol (this is the value that should be used for the estimation of endocannabinoid 2-AG); BDNF = brain-derived neurotrophic factor; DEA = docosatetraenoylethanolamide (novel endocannabinoid); OLA = oleamide (novel endocannabinoid).

## Data Availability

The datasets used and/or analyzed during the current study are available from the corresponding author upon reasonable request.

## Abbreviations

1-AG: 1-Arachidonoylglycerol
2-AG: 2-Arachidonoylglycerol
AEA: N-Arachidonoylethanolamine
ANOVA: Analysis of variance
BDNF: Brain-derived neurotrophic factor
BSP: Bhava Spandana Program
CB1: Cannabinoid receptor type 1
CES-D: Center for Epidemiological Studies Depression Scale
DEA: Docosatetraenoylethanolamide
eCB: Endocannabinoid
FAAH: Fatty acid amide hydrolase
IL: Interleukin
MAAS: Mindful attention and awareness scale
OLA: Oleoylethanolamide
PWB: Psychological well-being scale
THC: Tetrahydrocannabinol
TNF: Tumor necrosis factor.

## Supplementary Materials

Supplementary Materials Associations of changes in biomarkers (20% increase or not) with changes in psychological surveys.
