# Supplementary material for: Inner Engineering Practices and Advanced 4-day Isha Yoga Retreat Are Associated with Cannabimimetic Effects with Increased Endocannabinoids and Short-Term and Sustained Improvement in Mental Health: A Prospective Observational Study of Meditators
Source: Evid Based Complement Alternat Med. 2020 Jun 5;2020:8438272. doi: 10.1155/2020/8438272 (PMC7293737; doi:10.1155/2020/8438272)
Supplement: Supplementary Materials — Associations of changes in biomarkers (20% increase or not) with changes in psychological surveys. [file 8438272.f1.docx]

**Supplementary Table.** Associations of changes in biomarkers (20% increase or not) with changes in psychological surveys.

|  |  | **Biomarker Increase** | | **No Biomarker Increase** | |  |
| --- | --- | --- | --- | --- | --- | --- |
| **Biomarker** | **Psychological parameter** | **N** | **Mean (SD)** | **N** | **Mean (SD)** | ***P* value** |
| Anandamide | CES-D (Depression) | 79 | -2.70 (8.32) | 54 | -4.89 (10.51) | 0.183 |
| (ng/mL) | Anxiety Scale | 78 | -0.57 (0.71) | 54 | -0.56 (0.83) | 0.966 |
|  | MAAS (MINDFULNESS) | 79 | 0.77 (0.94) | 54 | 0.55 (0.81) | 0.163 |
|  | Happiness Score | 78 | 2.22 (1.95) | 54 | 2.02 (1.77) | 0.550 |
|  | PWB (Well-Being) Total | 78 | 18.05 (22.49) | 54 | 13.57 (20.37) | 0.245 |
|  | PWB Autonomy | 78 | 3.54 (5.37) | 54 | 2.11 (3.86) | 0.096 |
|  | PWB Environmental Mastery | 78 | 2.79 (4.93) | 54 | 1.94 (4.58) | 0.318 |
|  | PWB Personal Growth | 78 | 2.08 (4.22) | 54 | 1.54 (3.89) | 0.457 |
|  | PWB Positive Relations | 78 | 3.38 (5.73) | 54 | 3.54 (5.49) | 0.879 |
|  | PWB Purpose in Life | 78 | 1.79 (5.11) | 54 | 1.46 (4.21) | 0.694 |
|  | PWB Self-Acceptance | 78 | 4.46 (5.31) | 54 | 2.98 (5.59) | 0.126 |
| 2-AG | CES-D (Depression) | 95 | -3.77 (9.53) | 38 | -3.16 (8.79) | 0.734 |
| (ng/mL) | Anxiety Scale | 94 | -0.64 (0.76) | 38 | -0.39 (0.72) | 0.082 |
|  | MAAS (MINDFULNESS) | 95 | 0.78 (0.91) | 38 | 0.42 (0.82) | 0.031* |
|  | Happiness Score | 94 | 2.34 (1.78) | 38 | 1.63 (2.02) | 0.049* |
|  | PWB (Well-Being) Total | 94 | 19.00 (21.59) | 38 | 9.34 (20.59) | 0.020* |
|  | PWB Autonomy | 94 | 3.54 (4.98) | 38 | 1.50 (4.20) | 0.028* |
|  | PWB Environmental Mastery | 94 | 3.21 (4.92) | 38 | 0.55 (3.90) | 0.003* |
|  | PWB Personal Growth | 94 | 2.04 (4.17) | 38 | 1.39 (3.87) | 0.411 |
|  | PWB Positive Relations | 94 | 4.19 (5.45) | 38 | 1.61 (5.66) | 0.016* |
|  | PWB Purpose in Life | 94 | 1.54 (5.17) | 38 | 1.95 (3.51) | 0.659 |
|  | PWB Self-Acceptance | 94 | 4.47 (5.17) | 38 | 2.34 (5.92) | 0.042* |
| 1-AG | CES-D (Depression) | 122 | -3.49 (9.12) | 11 | -4.78 (11.52) | 0.659 |
| (ng/mL) | Anxiety Scale | 121 | -0.60 (0.75) | 11 | -0.26 (0.76) | 0.161 |
|  | MAAS (MINDFULNESS) | 122 | 0.69 (0.89) | 11 | 0.51 (0.97) | 0.512 |
|  | Happiness Score | 121 | 2.24 (1.87) | 11 | 1.00 (1.61) | 0.035* |
|  | PWB (Well-Being) Total | 121 | 17.36 (21.89) | 11 | 3.73 (14.72) | 0.045* |
|  | PWB Autonomy | 121 | 3.17 (4.89) | 11 | 0.64 (3.70) | 0.098 |
|  | PWB Environmental Mastery | 121 | 2.52 (4.88) | 11 | 1.64 (3.78) | 0.560 |
|  | PWB Personal Growth | 121 | 2.06 (4.06) | 11 | -0.36 (3.78) | 0.059 |
|  | PWB Positive Relations | 121 | 3.70 (5.63) | 11 | 0.64 (4.80) | 0.083 |
|  | PWB Purpose in Life | 121 | 1.69 (4.86) | 11 | 1.27 (3.32) | 0.779 |
|  | PWB Self-Acceptance | 121 | 4.21 (5.39) | 11 | -0.09 (4.72) | 0.012* |
| Total AG | CES-D (Depression) | 119 | -3.56 (9.22) | 14 | -3.91 (10.26) | 0.893 |
| (ng/mL) | Anxiety Scale | 118 | -0.61 (0.76) | 14 | -0.23 (0.68) | 0.079 |
|  | MAAS (MINDFULNESS) | 119 | 0.72 (0.88) | 14 | 0.30 (0.96) | 0.098 |
|  | Happiness Score | 118 | 2.28 (1.87) | 14 | 0.93 (1.49) | 0.010* |
|  | PWB (Well-Being) Total | 118 | 17.48 (21.95) | 14 | 5.57 (16.16) | 0.052 |
|  | PWB Autonomy | 118 | 3.18 (4.94) | 14 | 1.07 (3.56) | 0.124 |
|  | PWB Environmental Mastery | 118 | 2.60 (4.91) | 14 | 1.14 (3.51) | 0.283 |
|  | PWB Personal Growth | 118 | 2.04 (4.07) | 14 | 0.29 (4.01) | 0.129 |
|  | PWB Positive Relations | 118 | 3.82 (5.60) | 14 | 0.29 (4.89) | 0.025* |
|  | PWB Purpose in Life | 118 | 1.69 (4.90) | 14 | 1.43 (3.25) | 0.848 |
|  | PWB Self-Acceptance | 118 | 4.15 (5.36) | 14 | 1.36 (5.84) | 0.070 |
| DEA | CES-D (Depression) | 80 | -3.54 (8.46) | 53 | -3.67 (10.51) | 0.935 |
| (ng/mL) | Anxiety Scale | 80 | -0.59 (0.71) | 52 | -0.54 (0.83) | 0.696 |
|  | MAAS (MINDFULNESS) | 80 | 0.78 (0.94) | 53 | 0.52 (0.81) | 0.105 |
|  | Happiness Score | 80 | 2.20 (1.91) | 52 | 2.04 (1.83) | 0.630 |
|  | PWB (Well-Being) Total | 80 | 18.03 (22.04) | 52 | 13.44 (21.01) | 0.237 |
|  | PWB Autonomy | 80 | 3.30 (5.30) | 52 | 2.42 (4.03) | 0.311 |
|  | PWB Environmental Mastery | 80 | 2.89 (4.77) | 52 | 1.77 (4.78) | 0.191 |
|  | PWB Personal Growth | 80 | 2.10 (4.01) | 52 | 1.48 (4.21) | 0.397 |
|  | PWB Positive Relations | 80 | 3.71 (5.79) | 52 | 3.04 (5.37) | 0.502 |
|  | PWB Purpose in Life | 80 | 1.73 (4.85) | 52 | 1.56 (4.62) | 0.844 |
|  | PWB Self-Acceptance | 80 | 4.30 (5.60) | 52 | 3.17 (5.21) | 0.248 |
| OLA | CES-D (Depression) | 69 | -4.27 (10.11) | 64 | -2.86 (8.34) | 0.384 |
| (ng/mL) | Anxiety Scale | 68 | -0.62 (0.85) | 64 | -0.52 (0.65) | 0.458 |
|  | MAAS (MINDFULNESS) | 69 | 0.66 (0.93) | 64 | 0.70 (0.86) | 0.827 |
|  | Happiness Score | 68 | 2.22 (1.90) | 64 | 2.05 (1.86) | 0.596 |
|  | PWB (Well-Being) Total | 68 | 15.81 (20.01) | 64 | 16.66 (23.47) | 0.823 |
|  | PWB Autonomy | 68 | 2.78 (4.77) | 64 | 3.14 (4.95) | 0.670 |
|  | PWB Environmental Mastery | 68 | 2.81 (4.75) | 64 | 2.06 (4.84) | 0.373 |
|  | PWB Personal Growth | 68 | 1.76 (3.87) | 64 | 1.95 (4.33) | 0.792 |
|  | PWB Positive Relations | 68 | 3.78 (5.62) | 64 | 3.09 (5.63) | 0.485 |
|  | PWB Purpose in Life | 68 | 1.22 (4.95) | 64 | 2.13 (4.51) | 0.275 |
|  | PWB Self-Acceptance | 68 | 3.46 (4.74) | 64 | 4.28 (6.13) | 0.387 |
| BDNF | CES-D (Depression) | 71 | -3.05 (8.70) | 62 | -4.21 (9.96) | 0.476 |
| (pg/mL) | Anxiety Scale | 71 | -0.46 (0.74) | 61 | -0.69 (0.77) | 0.091 |
|  | MAAS (MINDFULNESS) | 71 | 0.69 (0.92) | 62 | 0.66 (0.87) | 0.860 |
|  | Happiness Score | 71 | 2.04 (1.82) | 61 | 2.25 (1.95) | 0.535 |
|  | PWB (Well-Being) Total | 71 | 17.00 (21.35) | 61 | 15.31 (22.20) | 0.657 |
|  | PWB Autonomy | 71 | 3.32 (4.85) | 61 | 2.52 (4.84) | 0.346 |
|  | PWB Environmental Mastery | 71 | 2.48 (4.93) | 61 | 2.41 (4.66) | 0.935 |
|  | PWB Personal Growth | 71 | 2.10 (4.00) | 61 | 1.57 (4.19) | 0.464 |
|  | PWB Positive Relations | 71 | 3.45 (5.08) | 61 | 3.44 (6.23) | 0.993 |
|  | PWB Purpose in Life | 71 | 1.99 (4.39) | 61 | 1.28 (5.14) | 0.395 |
|  | PWB Self-Acceptance | 71 | 3.66 (5.44) | 61 | 4.08 (5.51) | 0.661 |

Abbreviations:

1-AG = 1-arachidonoylglycerol (degradation product of 2-arachidonoylglycerol); 2-AG = arachidonoylglycerol (unstable in plasma without pH adjustment); Total AG = sum of 1-arachidonoylglycerol and 2-arachidonoylglycerol (this is the value that should be used for the estimation of endocannabinoid 2-AG); BDNF = Brain Derived Neurotrophic Factor; CES-D = Center for Epidemiologic Studies Depression; DEA = Dopamine ethanolamide (novel endocannabinoid); OLA = Oleamide (novel endocannabinoid); MAAS = Mindful Attention & Awareness Scale; PWB = Psychological Well-Being
